# Catastrophic Merkel Cell Carcinoma in a Liver Transplant Recipient

**DOI:** 10.7759/cureus.45133

**Published:** 2023-09-12

**Authors:** Rabab S Isa, Emily Clarke, Hanna Fanous, Anokhi Jambusaria-Pahlajani

**Affiliations:** 1 Department of Internal Medicine, Division of Dermatology, Dell Medical School at the University of Texas at Austin, Austin, USA

**Keywords:** nmsc(nonmelanoma skin cancer ), ­skin cancer, calcineurin inhibitor, mtor inhibitor, immunosuppression, transplant, merkel cell carcinoma

## Abstract

Merkel cell carcinoma (MCC) is a rare skin cancer, is difficult to diagnose, and carries a high mortality rate. Solid organ transplant recipients (SOTR) are at a disproportionately increased risk of MCC and other malignancies due to chronic immunosuppression. We discuss the case of a 47-year-old woman with a remote history of liver transplant on chronic immunosuppression with tacrolimus for over a decade who presented for a third recurrence of MCC on her left forearm. This case report underscores the importance of a risk-stratified approach to regular dermatologic care and skin cancer screening in this vulnerable population.

## Introduction

Merkel cell carcinoma (MCC) is a rare, highly aggressive cutaneous malignancy, arising from Merkel cells in the basal layer of the epidermis [1]. MCC carcinogenesis is associated with Merkel cell polyomavirus (*MCPyV*), immunosuppression, and chronic ultraviolet light exposure [2,3]. MCC has a mortality rate of 30%, twice that of malignant melanoma [4], and due to the rarity of the tumor, diagnosis may be delayed. Organ transplant recipients’ risk for MCC is 24 times higher than the general population due to chronic immunosuppression [1]. The objective of this case report is to increase awareness that Solid organ transplant recipients (SOTR) are at higher risk of developing skin cancers, including MCC. This case also reviews evidence-based guidelines for the diagnosis and management of MCC and highlights the importance of routine dermatologic care and skin cancer surveillance in SOTR to promote early detection of skin cancers.

## Case presentation

A 47-year-old woman with a history of type II diabetes mellitus, remote liver transplantation for chronic Hepatitis C induced cirrhosis, and the recent diagnosis of Merkel cell carcinoma (MCC) presented with acute left forearm pain due to a third MCC recurrence. Initial diagnosis of the MCC was made by excisional biopsy of the left forearm six months prior. She subsequently underwent excision with negative margins and lymph node dissection, which identified five positive nodes. The patient was switched from tacrolimus to sirolimus for immunosuppression at that time. Shortly after, she experienced her second recurrence and underwent her second resection with adjuvant radiation therapy. 

**Figure 1:**
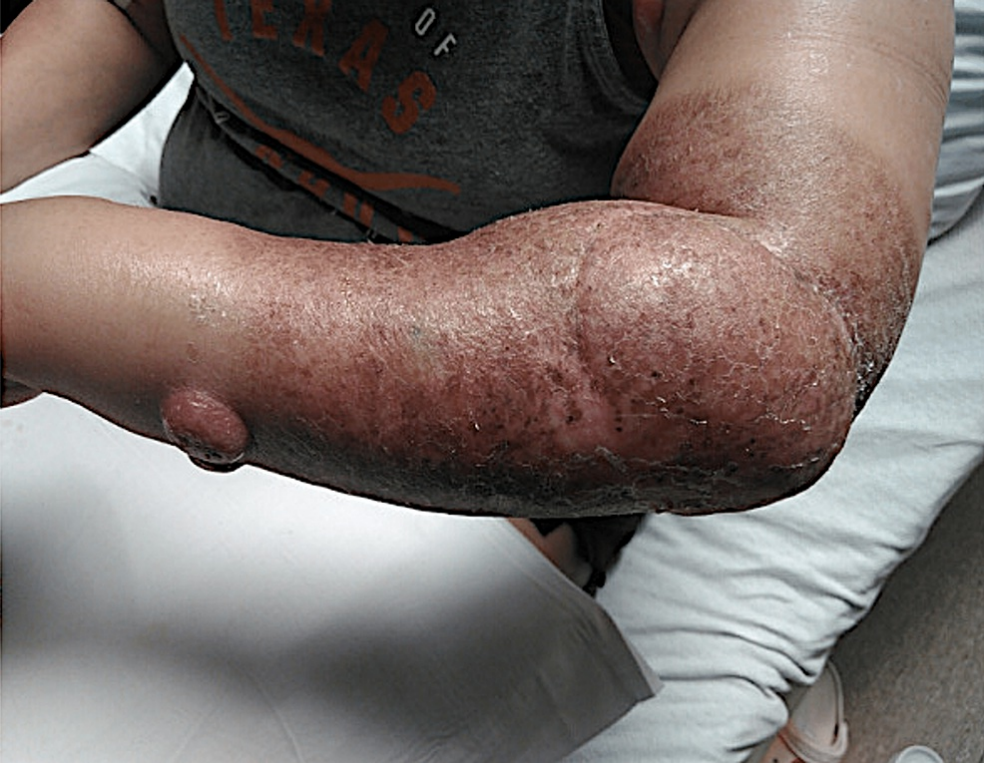
Ulcerated pink nodule measuring 2.5 cm on the left dorsal forearm.

**Figure 2:**
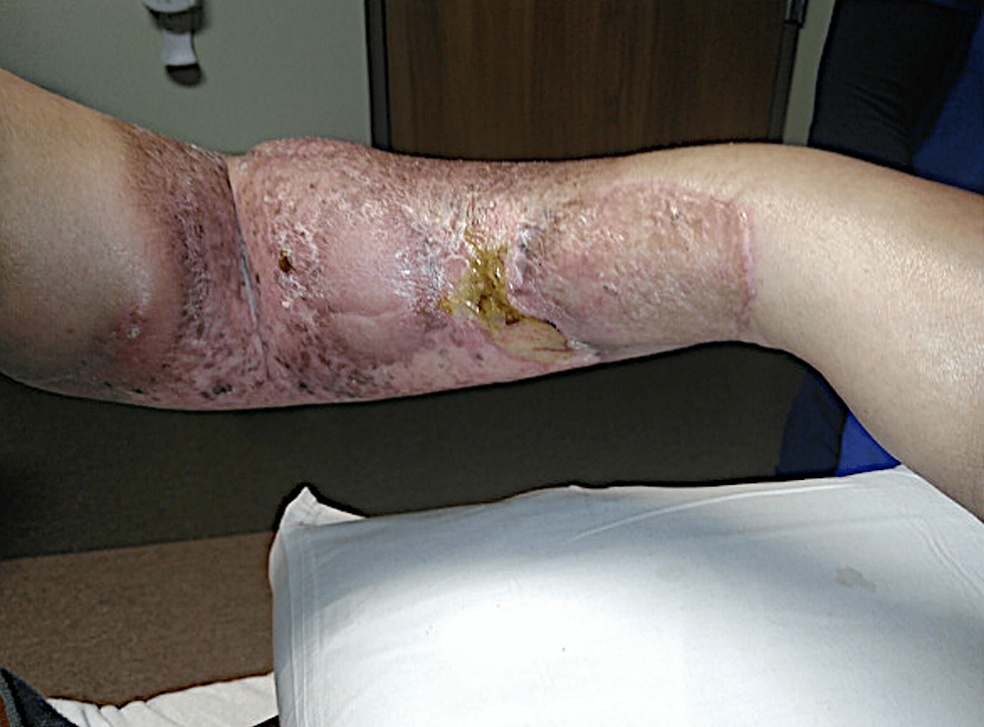
Ulcerated plaque with serosanguinous crust on the ventral surface of the left forearm.

On presentation to the hospital, the patient reported an enlarging skin nodule associated with pain and tingling of her left arm. On physical examination, there was an ulcerated pink nodule measuring 2.5 cm on the left dorsal forearm (Figure 1) and an ulcerated plaque with serosanguinous crust noted on the ventral forearm (Figure 2).

**Figure 3:**
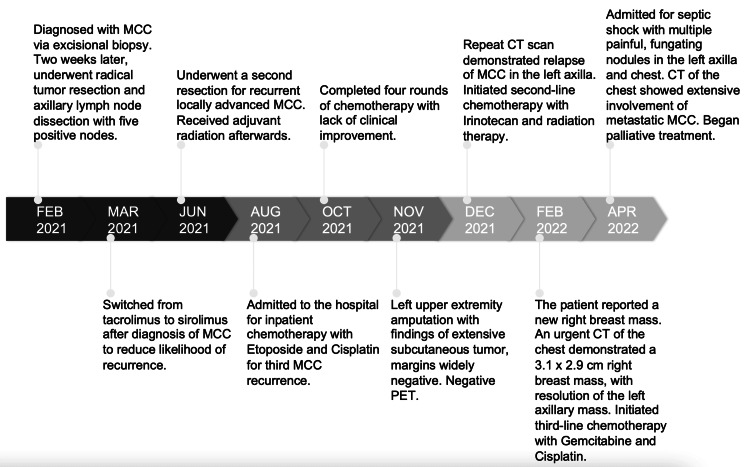
Historical and current information from this episode of care organized as a timeline.

Computed tomography (CT) scan of the chest, abdomen, and pelvis demonstrated no evidence of metastatic disease. Due to previous treatment failure of surgery and radiation therapy, systemic chemotherapy with etoposide and cisplatin was initiated with good tolerability, and the transplant team was able to further taper her sirolimus dose. However, despite four cycles of systemic chemotherapy, she continued to experience recurrent MCC skin lesions, pain, and dysfunction in the left upper extremity, ultimately resulting in left shoulder disarticulation and amputation. The amputation demonstrated findings of extensive subcutaneous MCC tumor, with positron emission tomography (PET) scan and margins widely negative. Shortly thereafter, she was found to have metastatic MCC of the left axilla and breast on imaging, which did not respond to radiation or second-or third-line chemotherapy. She ultimately returned with multiple painful, fungating nodules of MCC in the left axilla and chest, and CT demonstrated infiltrative MCC extensively throughout the chest. A decision was made to initiate palliative care given rapid MCC metastasis and recalcitrance to treatment. A complete timeline of events is detailed in Figure 3.

## Discussion

Merkel cell carcinoma (MCC), a rare and aggressive cutaneous malignancy, is more common in males with lower Fitzpatrick skin types, older age, immunosuppression, chronic sun exposure, and active malignancy [2]. It presents as a rapidly progressive, painless cutaneous or subcutaneous nodule that is often indurated, ulcerated, or violaceous [3]. Most MCC lesions are found in sun-exposed areas, particularly the head and neck (43%) and upper extremities (24%), but the presentation can be nonspecific, making initial diagnosis difficult [4]. Most patients (65%) present with only local manifestations of the disease [2], but up to 26% of patients have nodal involvement, and 8% have distant metastatic disease, most commonly to the skin, lung, bone, and brain [2,4].

Diagnosis of MCC is often delayed due to its low prevalence and nonspecific appearance, which can result in initial misdiagnosis as a benign skin lesion [2]. On histopathology, MCC is usually diagnosed with a skin biopsy, which characteristically demonstrates dermal sheets of small, round, blue cells with vesicular nuclei and little cytoplasm on hematoxylin and eosin (H&E) staining [3]. MCC stains positive for immunological markers such as CK20 and neuroendocrine markers such as chromogranin, synaptophysin, and CD56 [1,4].

The National Comprehensive Cancer Network (NCCN) guidelines for MCC recommend beginning with a complete skin and lymph node examination [5]. Clinicians should obtain a biopsy with H&E staining and immunopanel workup [5]. If the patient is immunosuppressed, the NCCN recommends reducing the immunosuppressive dose as appropriate. Quantification of *MCPyV* oncoprotein antibodies may be considered as part of the initial workup, as up to 80% of MCC cases may demonstrate positivity [5-7]. *MCPyV* seronegative patients may have a higher risk of recurrence, and a rising titer in seropositive patients may be an early indicator of recurrence [5,7]. Imaging is recommended when metastatic or unresectable MCC is suspected based on initial history and physical [5]. Sentinel lymph node biopsy (SLNB) is the most reliable staging tool and is recommended for MCC-positive patients with ≥1 baseline risk factors (larger primary tumor greater than one centimeter, chronic T-cell immunosuppression, HIV, chronic lymphocytic leukemia, SOTR, head/neck primary site, lymphovascular invasion) [2,5]. If SLNB is positive for MCC, imaging, multidisciplinary consultation, node dissection and/or radiotherapy to the nodal basin are recommended, along with consideration of clinical trials or neoadjuvant immunotherapy [5]. Dermatologic surveillance for recurrence is recommended every three to six months for three years and every six to 12 months thereafter, with imaging as clinically indicated [5]. If recurrence occurs, clinical trials, systemic therapy, radiotherapy, surgery, or supportive care can be considered [5]. Ongoing clinical trials targeting the PD-1/PDL-1 immune-checkpoint pathway with pembrolizumab and avelumab, in combination with CTLA-4 inhibition, have also shown promising results [3].

The link between MCC and immunosuppression is well-documented. MCC disproportionately affects immunosuppressed patients, including those with Human Immunodeficiency Virus (HIV), solid organ transplants, and autoimmune disease [1,2,5]. For transplant patients, pharmacologic agents that prevent graft rejection result in iatrogenic immunosuppression, increasing the risk for several variants of skin cancer including MCC. Calcineurin inhibitors such as tacrolimus, which were utilized in this patient’s case for over a decade, cause a 200-fold increase in skin cancer risk [8]. For this reason, recent studies advocate for the use of mechanistic target of rapamycin (mTOR) inhibitors, including sirolimus and everolimus, over the use of calcineurin inhibitors [9]. Utilization of mTOR inhibitors may reduce the incidence of recurrence and improve survival in patients with MCC and other post-transplant skin cancers [9]. Decreasing immunosuppressive regimens may also improve the clinical course of MCC [3]. In this case, the patient had a SOTR and was taking tacrolimus for over 10 years without receiving dermatologic care or routine skin cancer screenings. Her immunosuppressive regimen was changed from tacrolimus to a lower dose of sirolimus upon discharge to reduce recurrence.

Recent studies demonstrate that 14% of all solid organ transplant recipients in the United States will develop a form of skin cancer within 10 years of transplantation [10]. Skin cancer rates are greatest for those with thoracic organ transplants including lung transplants (3.52%) and heart transplants (1.63%) [11,12]. There is still risk, albeit lower, for patients with abdominal organ transplants, including liver (1.19%) [11]. Therefore, screening for skin cancer remains paramount in this population.

This case highlights a catastrophic course of MCC resulting in the ultimate need for palliative care and therapy. This patient case also reinforces the need for routine skin cancer screening with a risk-stratified approach to surveillance in solid organ transplant patients. All patients should undergo skin cancer screening after solid organ transplantation to assess baseline skin cancer risk. The SUNTRAC tool [13], which was developed based on five risk factors (white race, pre-transplant history of skin cancer, older age at the time of transplant, male sex, and history of thoracic organ transplant), stratifies patients into four risk groups and establishes optimal screening frequencies for patients and may assist with this. Patients who develop post-transplant skin cancers should be followed closely for recurrence.

## Conclusions

MCC is a rare skin cancer that can have catastrophic implications if not managed appropriately. This case demonstrates a unique example of MCC in a 47-year-old female patient with an underlying history of remote liver transplant on chronic immunosuppression with tacrolimus. This case report aims to increase awareness that SOTR are at higher risk of developing catastrophic skin cancers such as MCC, review evidence-based guidelines when treating a patient with suspected MCC, and highlight the importance of routine dermatologic care and skin cancer surveillance with a risk-stratified approach in SOTR to facilitate early detection of cutaneous malignancy.
